# Patient-reported outcomes after minimally invasive sacro-iliac joint surgery: a cohort study based on the Swedish Spine Registry

**DOI:** 10.2340/17453674.2024.40817

**Published:** 2024-06-14

**Authors:** Engelke Marie RANDERS, Thomas Johan KIBSGÅRD, Britt STUGE, Andreas WESTBERG, Freyr Gauti SIGMUNDSSON, Anders JOELSON, Paul GERDHEM

**Affiliations:** 1 Division of Orthopaedic Surgery, Oslo University Hospital, Oslo, Norway; 2 Institute of Clinical Medicine, University of Oslo, Oslo, Norway; 3 Västmanlands County Hospital, Västerås, Sweden; 4 Department of Orthopaedics, Örebro University Hospital, Örebro, Sweden; 5 School of Medical Sciences, Faculty of Medicine and Health, Örebro University, Örebro, Sweden; 6 Department of Surgical Sciences, Uppsala University, Uppsala ,Sweden; 7 Department of Orthopaedics and Hand surgery, Uppsala University Hospital, Uppsala, Sweden

## Abstract

**Background and purpose:**

There is conflicting evidence regarding treatment outcomes after minimally invasive sacroiliac joint fusion for long-lasting severe sacroiliac joint pain. The primary aim of our cohort study was to investigate change in patient-reported outcome measures (PROMs) after minimally invasive sacroiliac joint surgery in daily practice in the Swedish Spine Registry. Secondary aims were to explore the proportion of patients reaching a patient acceptable symptom score (PASS) and the minimal clinically important difference (MCID) for pain scores, physical function, and health-related quality of life outcomes; furthermore, to evaluate self-reported satisfaction, walking distance, and changes in proportions of patients on full sick leave/disability leave and report complications and reoperations.

**Methods:**

Data from the Swedish Spine Registry was collected for patients with first-time sacroiliac joint fusion, aged 21 to 70 years, with PROMs available preoperatively, at 1 or 2 years after last surgery. PROMs included Oswestry Disability Index (ODI), Numeric Rating Scale (NRS) for low back pain (LBP) and leg pain, and EQ-VAS, in addition to demographic variables. We calculated mean change from pre- to postoperative and the proportion of patients achieving MCID and PASS.

**Results:**

68 patients had available pre- and postoperative data, with a mean age of 45 years (range 25–70) and 59 (87%) were female. At follow-up the mean reduction was 2.3 NRS points (95% confidence interval [CI] 1.6–2.9; P < 0.001) for LBP and 14.8 points (CI 10.6–18.9; P < 0.001) for ODI. EQ-VAS improved by 22 points (CI 15.4–30.3, P < 0.001) at follow-up. Approximately half of the patients achieved MCID and PASS for pain (MCID NRS LBP: 38/65 [59%] and PASS NRS LBP: 32/66 [49%]) and physical function (MCID ODI: 27/67 [40%] and PASS ODI: 24/67 [36%]). The odds for increasing the patient’s walking distance to over 1 km at follow-up were 3.5 (CI 1.8–7.0; P < 0.0001), and of getting off full sick leave or full disability leave was 0.57 (CI 0.4–0.8; P = 0.001). In the first 3 months after surgery 3 complications were reported, and in the follow-up period 2 reoperations.

**Conclusion:**

We found moderate treatment outcomes after minimally invasive sacroiliac joint fusion when applied in daily practice with moderate pain relief and small improvements in physical function.

Minimally invasive sacroiliac joint surgery was introduced as a treatment for long-lasting severe sacroiliac joint pain in the early 2000s [1,2]. The literature on minimally invasive sacroiliac joint fusion is abundant, but only 3 RCTs exist [1,3-5]. These 3 RCTs show conflicting results regarding the efficacy of surgery in reducing pain and improving physical function [3-5]. 2 RCTs comparing surgery with conservative treatment found that surgery was superior to conservative treatment in reducing pain and increasing physical function at 6 months [3,5]. The third sham-controlled RCT could not prove that surgery was better than sham surgery at 6 months [4].

The primary aim of our cohort study was to investigate change in patient-reported outcome measures (PROMs) after minimally invasive sacroiliac joint surgery in daily practice in the Swedish Spine Registry. Secondary aims were to explore the proportion of patients reaching a patient acceptable symptom score and the minimal clinically important difference (MCID) for pain scores, physical function, and health-related quality of life outcomes. Furthermore, the study aimed to evaluate self-reported satisfaction, walking distance, and changes in proportions of patients on full sick leave/disability leave, and to report complications and reoperations.

## Methods

This study is based on prospectively collected data in the Swedish Spine Register (SweSpine).

SweSpine collects data from individuals who undergo surgical treatment for spinal disorders [6]. The proportion of operating clinics using SweSpine is 98% (46 of 47 spine units), the proportion of surgeries registered is 86%, and accuracy of registered diagnoses is 97% [6,7]. Diagnosis, type of surgical procedure, and complications during the hospital stay are recorded by the surgeon, as well as additional surgeries. Complications during inpatient stay are reported and defined by the surgeon. Complications during the first 3 months after surgery are reported by the patient at the 1-year follow-up. Reoperations are defined as additional procedures on the same level and laterality. New index surgeries are defined as additional surgeries on the contralateral side. At admission, and 1, 2, 5, and 10 years postoperatively, patients are asked to answer self-assessment health questionnaires, sent and retrieved by regular mail or digital means.

### Study cohort

We collected data from individuals who had undergone minimally invasive sacroiliac joint surgery. The most routinely used surgical technique for treating sacroiliac joint pain in Sweden in this period was minimally invasive joint fusion with titanium triangular implants (iFuse, SI Bone Inc, Santa Clara, CA, USA). Registry data was extracted on January 23, 2020. Additional follow-up data for the individuals included in this study was searched for on August 22, 2023.

To make certain that the PROMs reflected sacroiliac joint pain, and no other source of low back pain, we included only patients who had undergone sacroiliac joint surgery as their index surgery with no former lumbar or sacroiliac joint surgery reported either in SweSpine or by the individuals themselves. Individuals with PROMs available at baseline and 1 year postoperatively or later were included in this study.

Inclusion criteria were:

- sacroiliac joint fusion;
- age 21–70 years;
- Swedish personal identification number;
- PROMs available preoperative and 1 year postoperatively or later;
- sacroiliac joint fusion performed before January 23, 2020;
- not a participant in the sham-controlled RCT [4].

### Outcomes

Demographic data was submitted by the patients and included smoking status, walking capacity, work status, and sick leave. The PROMs used were the Oswestry Disability Index (ODI) version 2.1 (from 0 = no disability to 100 = maximum disability) [8], Numeric Rating scale (NRS) for low back and leg pain (both ranging from 0 = no pain to 10 = worst imaginable pain) [9], and EQ visual analog scale (EQ VAS; where 0 is worst imaginable health status and 100 is best health status) [10]. At the follow-ups additional questions were answered by the patients: satisfaction with treatment (categorized as “satisfied,” “uncertain,” or “dissatisfied”); global assessment on leg and back pain (categorized as “pain free,” “much better,” “somewhat better,” “unchanged,” and “worse”); self-reported walking distance (categorized as “< 100 m,” “100–500 m,” “0.5–1 km,” “> 1 km”) and whether patients were on current full sick leave or disability leave [9]. For patients who underwent bilateral surgery, the outcomes were retrieved from the longest follow-up (1 or 2 years) after the most recent sacroiliac joint surgery.

### Statistics

Data is presented as mean with 95% confidence interval of the mean (CI) or number (%) with CI of proportions. Categorical variables were analyzed with the Pearson chi-square test. Normality of data was tested and verified by visual check of histograms. Continuous variables were analyzed with Student’s t-test for independent samples or paired sample t-test. The change in the proportion of patients reporting a walking distance > 1 km was evaluated, as well as the change in proportions of patients reporting to be on full sick leave or disability leave. Odds ratio was calculated using a generalized equation logistic regression for repeated measurements with an unstructured within-group correlation structure.

We calculated the proportion of patients reaching MCID for NRS back pain (≥ 2) [11], NRS leg pain (≥ 2) [12], ODI (≥ 15) [13], and EQ VAS (≥ 12) [10]. In addition, for ODI, NRS back pain, and NRS leg pain we calculated the proportion of patients having more than 30% reduction in scores, and the proportion of patients with PASS value below the cut-off [11,14]. For NRS back pain and leg pain we chose a cut-off value for PASS ≤ 4 [15), and the PASS value of ODI values was set to ≤ 25 [16].

Missing data was considered to be missing at random [17]. A sensitivity analysis using a multiple imputation model was performed to evaluate the impact missing data would have on the results (see Supplementary data). Sensitivity analysis showed small differences between models, and therefore analyses based on a “Last Observation Carried Forward” (LOCF) model was presented in the current study (see Supplementary data).

The differences reported in the LOCF data set are between baseline and the last follow-up. In those patients where last follow-up was 1 year postoperatively, 1 year data was carried forward to 2 years postoperatively.

Non-responder analyses were undertaken comparing baseline data for those who responded to baseline and the 1 year or 2 years’ follow-up, and those who did not respond.

All data analysis was completed using SPSS (version 29; IBM Corp, Armonk, NY, USA) or STATA (StataCorp, College Station, TX, USA).

### Ethics, funding, data sharing, and disclosures

The current study has been prepared in accordance with the Strengthening the Reporting of Observational Studies in Epidemiology (STROBE) Statement. Informed consent is not required since SweSpine applies the opt-out method, but answering the questionnaire is voluntary. The Regional Ethical Review Board in Stockholm has approved the study (number 2018/1463-31). Data is available from the national Swedish Spine Register (SweSpine) after approval by the Swedish Ethical Review Authority and according to the regulations in the General Data Protection Regulation and the Swedish Patient Data Act.

EMR received a public grant to cover a research affiliation from Sophies Minde Ortopedi AS. PG was supported by the Center for Innovative Medicine (CIMED), Karolinska Institutet, Sweden and Uppsala University, Sweden. The authors have no conflicts of interest. Complete disclosure of interest forms according to ICMJE are available on the article page, doi: 10.2340/17453674.2024.40817

## Results

116 patients met the inclusion criteria but 48 were excluded due to missing PROMs (Figure). The 68 patients with data available at 1 or 2 years postoperatively had a mean age of 45 years (range 25–70 years), 59 (87%) were female, 55 of 67 (82%) patients had had back pain for more than 2 years, 31 of 66 (47%) patients were on full sick leave or disability leave, and 2 of 68 (3%) patients were retired. The patients were operated on at 3 different centers in Sweden. One center performed the majority (51 out of 68) of the procedures. Bilateral surgery had been completed before final follow-up in 15 of 68 patients (22%) (Table 1). Last follow-up data was 1 year in 19 patients and 2 years in 49 patients.

**Table 1:** Patient baseline characteristics (N = 68). Values are count/total number (%) unless otherwise specified

Age, mean (range)	45 (25–70)
BMI (SD)	25.3 (3.9)
Female sex	59/68 (87)
Bilateral surgery	15/68 (22)
Smokers	2/68 (3)
Employment status	
Worker compensation/sick leave	
100% because of back pain	18/66 (27)
part time because of back pain	10/66 (15)
yes, of other cause	1/66 (2)
none	37/66 (56)
Disability leave	
full time	13/65 (20)
part time	6/65 (9)
no	46/65 (71)
Retirement	2/68 (3)
Duration of symptoms	
Back pain	
1–2 years	12/67 (18)
> 2 years	55/67 (82)
Leg pain	
no pain	13/66 (20)
< 1 year	4/66 (6)
> 1 year	49/66 (74)
Medication	
Using painkillers/medication for back pain	
yes, regularly	45/66 (68)
yes, sometimes	16/66 (24)
no	5/66 (8)
Using opioids	
yes	18/38 (47)
no	16/38 (42)
don’t know	4/38 (11)

**Figure:**
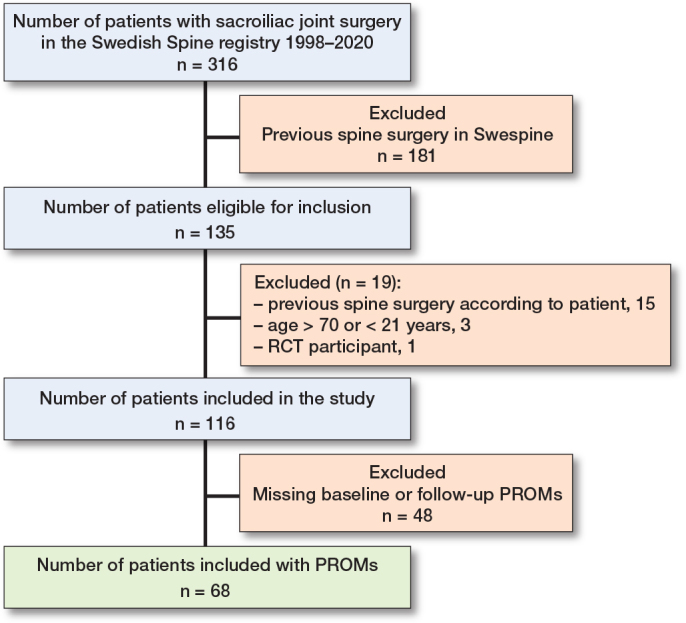
Flowchart of study inclusions and exclusions.

At last follow-up, the mean decrease in NRS LBP was 2.3 (CI 1.6–2.9; P < 0.001) (Table 2) and in NRS Leg pain 1.3 (CI 0.4–2.2; P = 0.01). 38 of 65 (59%, CI 46–70) patients had a reduction of more than 2 points in NRS LBP and 32 of 66 (49%, CI 36–60) reached the defined PASS value for NRS LBP of ≤ 4 (Table 3). Approximately half the patients reported more than 30% reduction in both NRS LBP (33 of 65 [51%, CI 39–63]) and NRS Leg pain (31 of 65 [48%, CI 36–60]) (Table 3).

**Table 2:** Outcome in regard to pain, physical function, and health-related quality of life

Factor	Number of patients	Preoperative mean (SD)	2-year follow-up mean (SD)	Difference mean (CI)	P value
NRS back pain	65	6.7 (1.9)	4.4 (2.6)	–2.3 (–2.9 to –1.6)	< 0.001
NRS leg pain	64	4.8 (2.7)	3.5 (3.0)	–1.3 (–2.2 to –0.4)	0.004
ODI	67	49.3 (12.6)	34.6 (34.6)	–14.8 (–18.9 to –10.6)	< 0.001
EQ5D VAS	63	39.1 (21.6)	61.9 (23.4)	22.8 (15.4 to 30.3)	< 0.001

NRS = Numeric Rating scale; ODI = Oswestry Disability Index;

EQ5D VAS = EuroQol 5 Dimension Visual Analogue Scale for perceived health status.

**Table 3:** Patients reaching patients acceptable symptom state, 30% improvement in outcome, and minimal clinically important difference

Factor	Patient acceptable symptom state (PASS)		30% improvement in outcome n/N (%) [CI of %]	Minimal clinically important difference (MCID)	
	Cut off value	Reaching PASS n/N (%) [CI of %]		Cut off value	Reaching MCID n/N (%) [CI of %]
ODI	≤ 25	24/67 (36) [25–48]	30/67 (45) [34–57]	≥ 15	27/67 (40) [29–52]
NRS back pain	≤ 4	32/66 (49) [36–60]	33/65 (51) [39–63]	≥ 2	38/65 (59) [46–70]
NRS leg pain	≤ 4	40/65 (62) [49–72]	31/65 (48) [36–60]	≥ 2	28/64 (39) [28–51]
EQ5D VAS	N/A	N/A	40/63 (63) [51–74]	≥ 12	39/63 (62) [50–73]

For abbreviations, see Table 2.

N/A = not available.

The mean reduction in ODI was 14.8 points (CI 10.6– 18.9; P < 0.001) (Table 2), and 27 of 67 (40%, CI 29–52) patients had a reduction of 15 points or more (Table 3). Only 24 of 67 (36%, CI 25–48) patients reached the PASS value for ODI of ≤ 25 (Table 3). The measure for health-related quality of life, EQ-VAS, showed similar slight improvement in perceived health state (Tables 2 and 3).

Preoperatively, 31 of 66 (47%) patients reported to be on full sick leave or full disability leave, which decreased to 21 of 66 (32%) patients at last follow-up (odds ratio 0.57, CI 0.40–0.80, P = 0.001).

Estimated walking distance was self-reported by the patients (Table 4). Before surgery 13 of 66 (21%) patients self-reported that they were able to walk more than 1 km, which increased to 32 of 66 (49%) patients at last follow-up (odds ratio 3.5, CI 1.8–7.0).

**Table 4:** Walking distance before and after sacroiliac joint fusion (N = 66). Values are count (%)

	Before the operation	After the operation
How long a walk can you do at normal pace?		
< 100 m	14 (21)	9 (14)
100–500 m	24 (36)	7 (11)
0.5–1 km	15 (23)	18 (27)
> 1 km	13 (21)	32 (49)

47 of 66 (71%, CI 59–81) patients reported to be satisfied with the result of surgery, whilst 19 of 66 (29%, CI 19–41) were uncertain or dissatisfied with the result. 30 of 67 (45%, CI 34–57) of patients reported they no longer had back pain or were much better, whilst 37 of 67 (55%, CI 43–67) patients were somewhat better, unchanged, or worse at last follow-up (Table 5).

**Table 5:** Self-reported satisfaction (N = 67). Values are count (%)

	Back pain	Leg pain
How is your pain compared with before the operation?		
Had no pain prior to surgery	0 (0)	14 (21)
Completely gone	4 (6)	10 (15)
Much better	26 (39)	16 (24)
Somewhat better	22 (33)	16 (24)
Unchanged	10 (15)	6 (9)
Worse	5 (8)	5 (8)

No complications were reported during the inpatient stay. Complications occurring during the first 3 months after surgery were 1 superficial wound infection, 1 deep vein thrombosis, and 1 reported leg weakness. Reoperations prior to 2 years follow-up were reported in 2 of 68 patients. 1 patient was reoperated on once and the other twice. All 3 operations were for repositioning of an implant used for the sacroiliac fixation.

There were no statistically significant differences between the responders (n = 68) and the non-responders (n = 48) regarding age (mean group difference 1.5, CI –1.9 to 2.3; P = 0.4) or sex (P = 0.97).

A sensitivity analysis to account for missing data using a multiple imputation model for all 116 patients identified to have undergone sacroiliac joint fusion did not produce results that differed substantially from the LOCF data results presented (see Supplementary data).

## Discussion

We aimed to investigate treatment outcomes after minimally invasive sacroiliac joint surgery in daily practice measured by PROMs in the Swedish Spine Registry.

We found that minimally invasive sacroiliac joint fusion used in daily practice by spine surgeons in Sweden gave moderate pain relief and small improvements in physical function, although to a lesser extent than most of the previous studies have shown [1,3-5]. Despite the moderate gain observed in the PROMs, the satisfaction with the procedure amongst patients was high.

There is much existing literature; however, only 3 former RCTs exist, which showed conflicting results [3-5]. The 2 unblinded RCTs, 1 from the United States and 1 from Europe, both showed minimally invasive surgery to be superior to nonoperative treatment [3,5]. The third, a double-blind sham-controlled RCT, could not prove minimally invasive sacroiliac joint surgery to be superior to sham surgery at 6 months [4]. The current study found similar improvements in pain (2.3 NRS points) to the surgically treated group in the double-blind sham-controlled RCT (2.6 NRS points), but somewhat better improvements in physical function (14 points ODI improvement versus 4 points in the sham-controlled RCT) [4]. Both the measured improvement in NRS LBP and physical function seen in the current study are substantially lower than what was observed in the surgically treated groups of the American RCT (5.3 NRS points reduction and 30 points ODI improvement) and the European RCT (4.3 NRS points reduction and 26 points ODI improvement) [3,5,18,19]. The percentage of patients who achieved MCID for NRS LBP (58%) was lower in the current study than in the American RCT (83%) and the European RCT (72%), but similar to the sham-controlled RCT (50%) [3-5,^18^,^19^]. Only 40% of patients reached MCID for ODI in the current study, much less than the American (68%) and European RCTs (64%) [3,5,18,19] . The observed differences in the measured treatment outcomes after surgery between these studies are interesting and might be influenced by several factors.

One such influencing factor could be the study population, which might differ between studies. The eligibility criteria used in the 3 RCTs are similar and reflect the existing consensus on how to diagnose and select patients for sacroiliac joint surgery [2-5]. Similar criteria were most likely used in daily practice to select surgical cases, making the study population in the current study comparable to the American, European, and sham-controlled RCTs. However, there are 3 differences that are interesting. First, there is a difference in sex distribution, with more females in the current study and the sham-controlled RCT (87% and 97%) [4]. Second, treatment of bilateralism of symptoms with consecutive surgeries led to a longer follow-up time in our study, as it did in the European and American RCTs [3,5]. By treating bilateralism of symptoms with bilateral surgery the observed pain relief would be expected to be larger. In addition, a longer follow-up time would have provided a longer rehabilitation period for improving both pain and physical function. Bilateral surgery and longer follow-up time combined could explain some of the differences observed in improvement of physical function in the current study and the American and European RCTs compared with the sham-controlled RCT [3-5]. Lastly, in the current study and the sham-controlled RCT there was a larger proportion of patients on sick leave or disability leave, patients who often have a poorer prognosis [3-5]. The reason for this higher proportion of patients on sick leave or disability leave might be the differences in social welfare systems between the countries in which the studies are completed. Although interesting, it is uncertain whether these slight differences between study populations are sufficient to explain the observed large differences between studies in measured treatment outcomes after sacroiliac joint surgery.

In comparison with the treatment outcomes measured through PROMS where only 49% reached the PASS for NRS LBP and only 36% the PASS for ODI, the patients reported high overall satisfaction with surgery at last follow-up. A large majority of patients (72%) reported being satisfied with the result of their surgery. There seems to be a discrepancy between the satisfaction the patients report themselves and the outcome in terms of PROMs. Both ODI and NRS are condition-specific PROMs for LBP but not specific for sacroiliac joint pain [20]. Condition-specific PROMs for LBP, which are widely used, might not be able to capture or reflect the important issues leading to improvement and hence patient satisfaction in this patient population after receiving surgical treatment for their sacroiliac joint pain – thus the PROMs’ so-called content validity might not be applicable [21]. Although facilitating easier comparison with other studies, the use of PROMs such as ODI, NRS, LBP, and EQ-VAS may have contributed to the observation of poorer treatment outcomes after surgery in our study compared with much of the former literature.

Patients afflicted by sacroiliac joint pain have a complex condition, and observed improvements in pain and physical function may be due to confounding factors other than the surgical treatment in itself [2]. Although the procedure is shown to have an effect on reducing pain and improving function, confounding factors that the patients are subjected to might influence the observed effectiveness of surgery [1,2,4]. Confounding factors might entail the natural course of the disease itself, nonoperative treatments such as physiotherapy, radiofrequency nerve ablations, and interdisciplinary treatment at rehabilitation centers [2]. Furthermore, meeting a healthcare provider who acknowledges the patient’s condition and who can offer surgical treatment has been described to be part of the placebo effect and might influence the result of surgery, as shown in a recent sham-controlled RCT [4,22]. The confounding factors mentioned above may have positively influenced the observed improvement seen in pain and physical function after sacroiliac joint surgery in our study from daily practice.

### Limitations

The sample size is small because we excluded patients with prior spine surgery. We believe that the patient group represents the patients with the highest potential for a good result after sacroiliac joint fusion, because all patients were operated on by experienced surgeons.

Another concern is that the responder rate was low, with follow-up data in 59% of patients. Based on earlier studies, non-responders may have poorer PROM outcomes but some have shown similar response to responders on PROMs [23-25]. Attrition bias may exist in our material and have influenced results. However, the sensitivity analysis performed using a multiple imputation technique model (elaborated in the Supplementary data) showed little difference between missing and non-missing data, indicating that the effect of attrition on outcome results is small, as supported by previous research [23,24].

### Conclusion

We found that minimally invasive sacroiliac joint fusion showed moderate improvement in PROMs regarding pain and physical function. There were only small changes in PROMs from baseline to follow-up. Only half of the patients reached MCID and PASS values for pain, physical function, and health-related quality of life. In contrast, patient satisfaction with surgery was high, many patients report improved walking distance, and fewer patients report being on full sick or disability leave at last follow-up.

In perspective, whether this improvement in treatment outcomes is large enough to defend the widespread use of sacroiliac joint surgery needs to be discussed in the medical community and researched further.

### Supplementary data

Sensitivity analyses are available as Supplementary data on the article page, doi: 10.2340/17453674.2024.40817

## Supplementary Material



## Notes

ER, TJK, and PG conceived the idea. PG, ER, and AJ retrieved data from SweSpine and verified the completeness of the data. TJK and ER did data analysis and statistical calculations. ER drafted the first version of the manuscript, and all authors contributed to revision and editing of the manuscript and tables.

The authors gratefully acknowledge the contribution of statistician Are Hugo Pripp, Oslo center of Biostatistics and Epidemiology, Research Support Services, Oslo University Hospital Oslo, for support with statistical analysis.

Handling co-editors: Ilkka Helenius and Robin Christensen

*Acta* thanks Samo Karl Fokter, Antti Malmivaara, and Marc J Nieuwenhuijse for help with peer review of this manuscript.
