# Supplementary material for: Patient-reported outcomes after minimally invasive sacro-iliac joint surgery: a cohort study based on the Swedish Spine Registry
Source: Acta Orthop. 2024 Jun 14;22:284–9. doi: 10.2340/17453674.2024.40817 (PMC11177861; doi:10.2340/17453674.2024.40817)
Supplement: Supplementary file 1 [file ActaO-95-40817-s1.pdf]

Supplementary data for

«Patient reported outcomes after sacroiliac joint surgery: a cohort study based on the Swedish Spine registry”

Table of contents

1. Missing data in Swespine 2

1.1 Structure of and data collection in Swespine 2

1.2 Missing data in Swespine 2

2. Handling of missing data in the current cohort study 2

2.1 Multiple imputation model for missing data 3

3. Tables 1 to 5 presenting baseline characteristics and outcome data based on Last Observation Carried Forward and Imputed data. 4

3.1 Baseline characteristics Table 1 4

3.1.1 **Table 1a.** Patient baseline characteristics. Data from the LOCF data set where 68 patients had available data at baseline. Values are count/total number (%) 4

3.1.2 **Table 1b.** Patient baseline characteristics. Analyses were done using a multiple imputation model and presents data of all 116 patients. Values are count/total number (%) 5

3.2 Tables presenting outcomes of pain, physical function and health related quality of life 6

3.2.1 **Table 2a.** Outcome on pain, physical function and health related quality of life. Complete case data with deleted missing data analysis by analysis 6

3.2.2 **Table 2b.** Outcome on pain, physical function and health related quality of life. Data shown as mean with (95%CI). Analysis based on last observation carried forward (1 year data are carried forward to 2 year) 6

3.2.3 **Table 2c.** Outcome in regard of pain, physical function and health related quality of life. Data shown as mean (95%CI). Analysis based on pooled multiple imputation technique data from an automatic imputation model with 20 imputations. N = 116 6

3.3 Tables showing proportion of patients reaching patients acceptable symptom state, 30% increase in outcome and minimal clinical important difference 7

3.3.1 **Table 3a.** Proportion of patients reaching patients acceptable symptom state, 30% increase in outcome, and minimal clinical important difference. Complete case data with missing data deleted analysis by analysis 7

3.3.2 **Table 3b**. Proportion of patients reaching patients acceptable symptom state, 30% increase in outcome and minimal clinical important difference. Analysis based on LOCF method, N = 68 7

3.3.3 **Table 3c**. Proportion of patients reaching patients acceptable symptom state, 30% increase in outcome and minimal clinical important difference. Analysis on imputed data from multiple imputation model 7

3.4 Tables showing walking distance before and after sacroiliac joint fusion 8

3.4.1 **Table 4a**. Walking distance before and after sacroiliac joint fusion. Complete case analysis with missing data deleted analysis by analysis 8

3.4.2 **Table 4b.** Walking distance before and after sacroiliac joint fusion. Analysis from LOCF method 8

3.4.3 **Table 4c.** Walking distance before and after sacroiliac joint fusion. Analysis on imputed data from multiple imputation model 8

3.5 Self-reported satisfaction tables 9

3.5.1 **Table 5a.** Self-reported satisfaction reported as n (%), N = 51. Complete case analysis 9

3.5.2 **Table 5b.** Self-reported satisfaction reported as n (%), N = 67. Analysis on LOCF method 9

3.5.3 **Table 5c.** Self-reported satisfaction reported as n (%), N = 116. Analysis of imputed data from multiple imputation model. 9

4. Reflections on the sensitivity analysis 10

References 10

1. Missing data in Swespine

1.1 Structure of and data collection in Swespine

Data is collected into Swespine through an opt-out method where questionnaires with PROMs (NRS, ODI, EQ-VAS and more) and demographic questions (age, sex, work status, walking distance, satisfaction, medication, etc.) are sent the patients by post [1]. Both filling out the questionnaires and returning them is voluntary [1]. All preoperative baseline data and postoperative follow-up data is mainly patient based [1]. Therefore, the availability of the data in Swespine is dependent on the patient´s willingness to fill out and return questionnaires. The surgical data (related to the operation) are the only data completed by the surgeon at the time of discharge from the hospital including diagnosis, procedure, implant, hospitalization time, antibiotic prophylaxis and occurrence of complications [1]. Any reoperations or complications must be reported by the surgeon and are dependent on the compliance and willingness of the surgeon to supply data into Swespine [1].

1.2 Missing data in Swespine

When a sample of patients with a certain procedure are extracted from Swespine for analysis, there will be various amounts of missing data due to the manner Swespine is organized and data is collected. The data will be missing at random and cannot be ignored when doing statistical analysis.

2. Handling of missing data in the current cohort study

Missing data can be "missing completely at random", which are the only kind of missing data that can be ignored, or data can be "missing at random" [2,3]. When data is "missing at random" certain statistical techniques can be used to handle such missing data. In the current cohort study, we chose to use a multiple imputation model to evaluate the influence of missing data on the results (Tables 1b, 2c, 3c, 4c and 5c). Results obtained from the multiple imputation model were compared to the results found by using a "Last Observation Carried Forward" (LOCF) method. The LOCF data contained 68 patients, where 1 year data was carried forward to 2 years in the cases where 2-year data were missing (Tables 1a, 2b, 3b, 4b and 5b). In addition, a complete case analysis was done (where all missing data were

deleted analysis by analysis) to evaluate the difference in results between the three methods. Results obtained from the complete case analysis are presented in Tables 2a, 3a, 4a and 5a.

2.1 Multiple imputation model for missing data

A multiple imputation model was used to complete missing data for the identified 116 patients who had undergone sacroiliac joint fusion and who fulfilled the inclusion criteria for the current study [2,3]. A multiple imputation with 20 imputations was done using SPSS [2]. Complete variables at baseline were used as predictors for imputation and consisted of age, sex and operating center. In addition, all variables with missing data were used to run the imputation model. The multiple imputation model was executed using default settings and adding appropriate clinical and/or scale limits to each missing variable. The imputed data sets were used for analysis to obtain pooled analysis results. The analyses resulting from pooled imputed data are presented in Tables 1b, 2c, 3c, 4c and 5c.

3. Tables 1 to 5 presenting baseline characteristics and outcome data based on Last Observation Carried Forward and Imputed data

3.1 Baseline characteristics Table 1

3.1.1 **Table 1a.** Patient baseline characteristics. Data from the LOCF data set where 68 patients had available data at baseline. Values are count/total number (%) unless otherwise specified

Age, mean (range)	45 (25–70)
BMI (SD)	25.3 (3.9)
Female sex	59/68 (87)
Bilateral surgery	15/68 (22)
Smokers	2/68 (3)
**Employment status**	
• Worker compensation/sick leave	
○ 100% because of back pain	18/66 (27)
○ Part time because of back pain	10/66 (15)
○ Yes, of other cause	1/66 (2)
○ None	37/66 (56)
• Disability leave	
○ Full time	13/65 (20)
○ Part time	6/65 (9)
○ No	46/65 (71)
• Retirement	2/68 (3)
**Duration of symptoms**	
• Back pain	
○ 1–2 years	12/67 (18)
○ >2 years	55/67 (82)
• **Leg pain**	
○ No pain	13/66 (20)
○ <1 year	4/66 (6)
○ >1 year	49/66 (74)
**Medication**	
• Using painkillers/medication for back pain	
○ Yes, regularly	45/66 (68)
○ Yes, sometimes	16/66 (24)
○ No	5/66 (8)
• Using opioids	
○ Yes	18/38 (47)
○ No	16/38 (42)
○ Don´t know	4/38 (11)

3.1.2 **Table 1b.** Patient baseline characteristics. Analyses were done using a multiple imputation model and presents data of all 116 patients. Values are count/total number (%) unless otherwise specified

Age, mean (range)	44 (24–70)
BMI (SD)	25.5 (4.2)
Female sex	103/116 (89)
Bilateral surgery	23/116 (20)
Smokers	20/116 (17)
**Employment status,**	
• Worker compensation/sick leave	
○ 100% because of back pain	32/116 (28)
○ Part time because of back pain	18/116 (15)
○ Yes, of other cause	15/116 (13)
○ None	51/116 (44)
• Disability leave	
○ Full time	25/116 (21)
○ Part time	19/116 (16)
○ No	73/116 (62)
• Retirement	17/116 (15)
**Duration of symptoms**	
• Back pain	
○ 1–2 years	28/116 (24)
○ >2 years	88/116 (75)
• Leg pain	
○ No pain	17/116 (15)
○ <1 year	24/116 (21)
○ >1 year	75/116 (64)
**Medication**	
• Using painkillers/medication for back pain	
○ Yes, regularly	68/116 (68)
○ Yes, sometimes	28/116 (24)
○ No	20/116 (17)
• Using opioids	
○ Yes	52/116 (45)
○ No	36/116 (31)
○ Don´t know	28/116 (24)

3.2 Tables presenting outcomes of pain, physical function and health related quality of life

3.2.1 **Table 2a.** Outcome on pain, physical function and health related quality of life. Complete case data with deleted missing data analysis by analysis

	Number of patients	Preoperative mean (SD)	2-year FU mean (SD)	Difference mean (CI)	P value
NRS back pain	47	7.0 (1.8)	4.2 (2.6)	–2.8 (–3.5 to –2.1)	<0.001
NRS leg pain	46	5.2 (2.8)	3.7 (2.9)	–1.5 (–2.5 to –0.5)	0.004
ODI	48	50.4 (12.5)	33.5 (22.8)	–16.9 (–21.9 to –11.8)	<0.001
EQ5D VAS	46	38.6 (21.5)	63.9 (24.2)	25.3 (16.0 to 34.5)	<0.001

FU: Follow-up: NRS: Numeric Rating scale; ODI = Oswestry Disability Index; EQ5D VAS= EuroQol 5 Dimension Visual Analogue

3.2.2 **Table 2b.** Outcome on pain, physical function and health related quality of life. Data shown as mean with (SD) or (CI). Analysis based on last observation carried forward (1 year data are carried forward to 2 year)

	Number of patients	Preoperative mean (SD)	2-year FU mean (SD)	Difference mean (CI)	P value
NRS back pain	65	6.7 (1.9)	4.4 (2.6)	–2.3 (–2.9 to –1.6)	<0.001
NRS leg pain	64	4.8 (2.7)	3.5 (3.0)	–1.3 (–2.2 to –0.4)	0.004
ODI	67	49.3 (12.6)	34.6 (34.6)	–14.8 (–18.9 to –10.6)	<0.001
EQ5D VAS	63	39.1 (21.6)	61.9 (23.4)	22.8 (15.4 to 30.3)	<0.001

For Abbreviations, see Table 2a.

3.2.3 **Table 2c.** Outcome in regard of pain, physical function and health related quality of life. Data shown as mean (CI). Analysis based on pooled multiple imputation technique data from an automatic imputation model with 20 imputations. N = 116

	Preoperative mean (SD)	1-year FU mean (SD)	2-year FU mean (SD)	Difference mean (CI)	P value
NRS back pain	6.8 (1.9)	4.8 (2.8)	4.5 (2.6)	–2.3 (–3.1 to –1.4)	<0.001
NRS leg pain	5.2 (2.6)	3.5 (3.5)	4.3 (2.8)	–0.9 (–1.8 to 0.05)	0.06
ODI	49.6 (12.5)	36.3 (19.2)	36.4 (20.7)	–13.1 (–18.5 to –7.8)	<0.001
EQ5D VAS	35.5 (23.4)	59.1(22.2)	61.0 (22.3)	25.5 (17.6 to 33.4)	<0.001

For Abbreviations, see Table 2a

3.3 Tables showing proportion of patients reaching patients acceptable symptom state, 30% improvement in outcome and minimal clinical important difference

3.3.1 **Table 3a.** Proportion of patients reaching patients acceptable symptom state, 30% improvement in outcome, and minimal clinical important difference. Complete case data with missing data deleted analysis by analysis

	Patient acceptable symptom state (PASS)		30% improvement in outcome	Minimal clinical important difference (MCID)	
	Cut off value	Reaching PASS n/N (%)	n/N (%)	Cut off value	Reaching MCID n/N (%)
ODI	≤25	19/51 (37)	24/48 (50)	≥15	24/48 (50)
NRS back pain	≤4	27/51 (52)	28/47 (60)	≥2	31/47 (66)
NRS leg pain	≤4	30/51 (58)	22/44 (55)	≥2	24/46 (48)
EQ5D VAS	N/A	N/A	30/46 (65)	≥12	31/46 (68)

For Abbreviations, see Table 2a and N/A = Not available.

3.3.2 **Table 3b**. Proportion of patients reaching patients acceptable symptom state, 30% increase in outcome and minimal clinical important difference. Analysis based on LOCF method, N = 68

	Patient acceptable symptom state (PASS)		30% improvement in outcome	Minimal clinical important difference (MCID)	
	Cut off value	Reaching PASS n/N (%) [CI of %]	n/N (%) [CI of %]]	Cut off value	Reaching MCID n/N (%) [CI of %]]
ODI	≤25	24/67 (36) [25–48]	30/67 (45) [34–57]	≥15	27/67 (40) [29–52]
NRS back pain	≤4	32/66 (49) [36–60]	33/65 (51) [39–63]	≥2	38/65 (59) [46–70]
NRS leg pain	≤4	40/65 (62) [49–72]	31/65 (48) [36–60]	≥2	28/64 (39) [28–51]
EQ5D VAS	N/A	N/A	40/63 (63) [51–74]	≥12	39/63 (62) [50–73]

For Abbreviations, see Table 2a and N/A = Not available.

3.3.3 **Table 3c**. Proportion of patients reaching patients acceptable symptom state, 30% improvement in outcome, and minimal clinical important difference. Analysis on imputed data from multiple imputation model

	Patient acceptable symptom state (PASS)		30% improvement in outcome	Minimal clinical important difference (MCID)	
	Cut off value	Reaching PASS n/N (%)	n/N (%)	Cut off value	Reaching MCID n/N (%)
ODI	≤25	37/116 (32)	53/116 (45)	≥15	52/116 (45)
NRS back pain	≤4	57/116 (49)	61/116 (53)	≥2	64/116 (55)
NRS leg pain	≤4	62/116 (54)	52/116 (45)	≥2	72/116 (62)
EQ5D VAS	N/A	N/A	74/116 (64)	≥12	79/116 (68)

For Abbreviations, see Table 2a and N/A = Not available.

3.4 Tables showing walking distance before and after sacroiliac joint fusion

3.4.1 **Table 4a**. Walking distance before and after sacroiliac joint fusion. Complete case analysis with missing data deleted analysis by analysis

How long walks can you do with normal pace?	Before the operation n/N (%)	After the operation n/N (%)
< 100 m	19/45 (24)	7/50 (14)
100–500 m	25/45 (31)	5/50 (10)
0.5–1 km	18/45 (23)	10/50 (20)
> 1 km	18/45 (23)	28/50 (56)

3.4.2 **Table 4b.** Walking distance before and after sacroiliac joint fusion. Analysis from LOCF method

How long walks can you do with normal pace?	Before the operation n/N (%)	After the operation n/N (%)
< 100 m	14/66 (21)	9/66 (14)
100–500 m	24/66 (36)	7/66 (11)
0.5–1 km	15/66 (23)	18/66 (27)
> 1 km	13/66 (21)	32/66 (49)

3.4.3 **Table 4c.** Walking distance before and after sacroiliac joint fusion. Analysis on imputed data from multiple imputation model

How long walks can you do with normal pace?	Before the operation n/N (%)	After the operation n/N (%)
< 100 m	28/116 (24)	28/116 (24)
100–500 m	32/116 (28)	24/116 (21)
0.5–1 km	28/116 (24)	22/116 (19)
> 1 km	28/116 (24)	43/116 (37)

3.5 Self-reported satisfaction tables

3.5.1 **Table 5a.** Self-reported satisfaction reported as n (%), N = 51. Complete case analysis

How is your pain compared with before the operation?	Back pain n (%)	Leg pain n (%)
Had no pain prior to surgery	0 (0)	9 (18)
Completely gone	4 (8)	7 (14)
Much better	23 (45)	11 (21)
Somewhat better	15 (29)	15 (30)
Unchanged	5 (10)	4 (8)
Worse	4 (8)	5 (10)

3.5.2 **Table 5b.** Self-reported satisfaction reported as n (%), N = 67. Analysis on LOCF method

How is your pain compared with before the operation?	Back pain n (%)	Leg pain n (%)
Had no pain prior to surgery	0 (0)	14 (21)
Completely gone	4 (6)	10 (15)
Much better	26 (39)	16 (24)
Somewhat better	22 (33)	16 (24)
Unchanged	10 (15)	6 (9)
Worse	5 (8)	5 (8)

3.5.3 **Table 5c.** Self-reported satisfaction reported as n (%), N = 116. Analysis of imputed data from multiple imputation model.

How is your pain compared with before the operation?	Back pain n (%)	Leg pain n (%)
Had no pain prior to surgery	0 (0)	15 (13)
Completely gone	17 (15)	28 (24)
Much better	37 (32)	19 (16)
Somewhat better	24 (21)	26 (22)
Unchanged	15 (13)	15 (13)
Worse	23 (20)	14 (12)

4. Reflections on the sensitivity analysis

Analysis in the current paper was done using "Last Observation Carried Forward" (LOCF) based on the assumption that there was no large further improvement expected from 1 to 2 years postoperative. The means for major treatment outcomes at 1- and 2-years follow-up (ODI, NRS back pain, NRS Leg pain and EQ VAS) were compared by paired sampled T-tests for those patients who had outcomes available at 1 and 2 years. This analysis showed small and statistically unsignificant improvements in NRS back pain (mean difference –0.8; CI –1.6 to –0.0, P = 0.04) and leg pain (mean difference –1; CI –1.9 to –0.3; P = 0.01) from 1 to 2 years. For ODI and EQ-VAS there was a small added improvement from 1 to 2 years which was not regarded as clinically important (respectively mean difference in ODI of –2.9 points (CI –7.4 to 1.6, P = 0.2) and for EQ-VAS 3.2 points (CI –2.4 to 8.8, P = 0.3). The current analysis provides information that this assumption appears reasonable. Furthermore, the sensitivity analysis done with a multiple imputation model shows little differences from analysis done on a LOCF data set. Based on both the sensitivity analysis and the assumption tested with paired t-tests, the results obtained from analysis on LOCF data sets does not seem to produce treatment outcomes that are either overestimated or underestimated. Therefore, it does not seem unreasonable to present results obtained from the LOCF data set in the current cohort study.

References

1. **Strömqvist B, Fritzell P, Hägg O, Jönsson B, Sandén B.** Swespine: the Swedish spine register: The 2012 report. Eur Spine J 2013; 22(4): 953-74. doi: 10.1007/s00586-013-2758-9.
2. **Austin P C, White I R, Lee D S, van Buuren S.** Missing data in clinical research: a tutorial on multiple imputation. Can J Cardiol 2021; 37(9): 1322-31. doi: 10.1016/j.cjca.2020.11.010.
3. **Christensen R, Ranstam J, Overgaard S, Wagner P.** Guidelines for a structured manuscript: statistical methods and reporting in biomedical research journals. Acta Orthop 2023; 94: 243-9. doi: 10.2340/17453674.2023.11656.
